# Role of cytokine therapy for renal cell carcinoma in the era of targeted agents

**DOI:** 10.3747/co.v16i0.417

**Published:** 2009-05

**Authors:** R. Koneru, S.J. Hotte

**Keywords:** Renal cancer, interferon, interleukin-2, cytokines, therapy

## Abstract

Starting in the late 1980s, cytokines were considered the mainstay of treatment for locally advanced or metastatic renal cell carcinoma (rcc) because of a lack of improved survival with either chemotherapy or hormonal therapy alone. The cytokine agents interferon alfa (ifnα) and interleukin-2 (il-2) have been the most evaluated, but a low overall response rate and a marginal survival advantage, coupled with significant toxicity, make these therapies less than ideal. Although complete tumour responses have occasionally been seen with high-dose il-2, this therapy is associated with significant morbidity and mortality, and its approval has been based on limited nonrandomized evidence. Newer anti-angiogenesis agents have been evaluated as single agents and in combination with infα, and these are now considered the standard of care for most patients with rcc. However, cytokines may still occasionally be recommended when angiogenesis inhibitors are not available or are contraindicated. In the present paper, we discuss the evidence for the use of cytokine therapy in the setting of pre– and post–targeted therapy for rcc.

## 1. INTRODUCTION

Before the advent of the new anti-angiogenic agents, cytokines were considered the mainstay of treatment for locally advanced or metastatic renal cell carcinoma (rcc) because of a lack of improved survival with either chemotherapy 1 or hormonal therapy alone. Spontaneous tumour regressions and the presence of an antitumour immune response in patients with rcc prompted the use of immunotherapy 2.

Like hormones and neurotransmitters, cytokines are a category of signalling molecules; they are used extensively in cellular communication. They can be proteins, peptides, or glycoproteins. Historically, however, the term “cytokine” has been used to refer to the immunomodulating agents such as the interleukins (ils) and interferons (infs), interferon alfa (infα) and il-2 being the most evaluated cytokine agents.

## 2. DISCUSSION

### 2.1 Interferon

Interferons are naturally occurring glycoproteins that have strong antiviral activity and the ability to modulate immune response and cell proliferation. The infs come in three subtypes: infα, infβ, and infγ 3. The antitumour activity of inf is mediated by various mechanisms—immunomodulation, antiproliferative activity, inhibition of angiogenesis, regulation of differentiation, interaction with growth factors, and modulation of gene expression, among others 4. The most extensively studied is infα, which has shown efficacy in rcc. Studies evaluating infγ have been negative.

In a Cochrane meta-analysis, treatment with infα was compared with treatment with a non-infα control 5,6. The pooled results of four randomized control trials (rcts) showed that infα was associated with greater remission rates [partial (pr) and complete (cr) responses] as compared with controls (medroxyprogesterone acetate or vinblastine). The pooled response rates were 12.5% for infα and 1.5% for controls, with a pooled odds ratio (or) of 7.61 [95% confidence interval (ci): 3.02 to 19.2]. Treatment with infα was associated with reduced 1-year mortality (or: 0.56; 95% ci: 0.40 to 0.77). A subgroup analysis that compared studies using the recombinant subtypes infα2a and infα2b showed no difference between the two subtypes in either objective response or 1-year survival.

A meta-analysis by Wirth 7 of 1042 patients found that the overall proportion of responses (crs and prs) to infα was 12%. The crs were rare. In patients with prior nephrectomy and in those with lung metastases, the proportion of objective responses was as high as 44%. The average time from the start of treatment to an objective response was about 3–4months. Metastases of the central nervous system tended not to respond to interferon, and soft-tissue disease tended to respond more readily than did metastases to bone.

A recent systematic review by the Cancer Care Ontario Program in Evidence-Based Care [cco-pebc (Canil C, Hotte S. Interferon-alfa in the treatment of patients with inoperable locally advanced or metastatic renal cell cancer, a clinical practice guideline. In preparation)] included eight randomized control trials 8–16 that directly evaluated the use of infα in locally advanced or metastatic rcc. These trials compared infα alone or in combination with other agents against control therapies considered to have little or no activity in rcc. The overall hazard ratio (hr) for death was 0.79, indicating a 21% reduction in the risk of death for patients treated with infα. The odds of objective response for patients receiving infα-containing regimens (4.4%–20%) were almost 7 times those for patients in control groups (0%–3%). Overall, toxicity appeared to be worse with infα than with non-infα therapy. The most common adverse events after 12 weeks of treatment with infα were anorexia, fatigue, dry mouth, and rigors. No toxic deaths were reported.

Doses and modality of administration of infα varied across the trials. It is unclear whether infα has a dose–response effect; however, it is likely that toxicity depends on dose and schedule. In view of this understanding, it was the consensus of the authors of the review that use of the dose and schedule from the largest rct showing benefit is reasonable 8,9. That trial gave an initial subcutaneous dose of 5×10^6^ IU, followed by 10×10^6^ IU subcutaneously on a thrice-weekly schedule for a total of 12 weeks unless disease progresses or an objective response is obtained. Treatment may be continued after 12 weeks in responding patients.

### 2.2 Interferon After Cytoreductive Surgery

The cco-pebc performed a meta-analysis of two randomized controlled trials comparing cytoreductive nephrectomy and infα2b with infα2b alone in patients with metastatic rcc 17. Overall survival (os) and response were assessed. The infα2b was initiated within 1 month of nephrectomy, was escalated to a subcutaneous dose of 5×10^6^ IU/m^2^ thrice weekly, and was continued until disease progression or completion of 52 weeks of therapy. In both trials, responses to infα2b were not significantly different between the trial arms. The pooled response rates were 6.9% for nephrectomy with infα2b and 5.7% for infα2b alone (*p* = 0.60). The pooled median survival time for patients treated with nephrectomy and infα2b was 13.6 months as compared with 7.8 months for patients treated with infα2b alone (*p* = 0.002). Nephrectomy was associated with a 31% lower risk of death (pooled hr: 0.69; 95% ci: 0.55 to 0.87). A survival advantage was maintained across all 3 stratification variables, which included performance status, site of metastases, and disease measurability. However, the magnitude of benefit seemed to greater for patients with a performance status of 0 as compared with 1 (28% vs. 22%), non-measurable as compared with measurable disease (51% vs. 25%), and lung-only as compared with not lung-only metastatic disease (37% vs. 30%). Combined therapy with nephrectomy and infα2b was well tolerated by most patients. Overall, the data support the recommendation that nephrectomy be considered in all patients fit enough to undergo the procedure.

### 2.3 Interleukin-2

The antitumour activity of the il-2 T-cell growth factor protein is not completely understood, but is believed to occur at least in part by direct activation of lymphoid cells. The il-2 affects proliferation and maturation of effector cells, enhancing the function of natural killer T cells, generating lymphokine-activated killer cells, and stimulating T-cell and B-cell growth, resulting in a reduction in tumour growth. The il-2 has no direct antitumour activity 18. Administration of il-2 can use any of three routes: high-dose il-2 bolus, continuous intravenous infusion, or subcutaneous injection.

A recent systematic review conducted by Hotte *et al.* 19 included rcts or meta-analyses of rcts comparing treatments with il-2 against regimens without il-2 in patients with unresectable or metastatic rcc and reporting data on at least one of the following outcomes: survival [os or progression-free survival (pfs), or time to progression], response rates, toxicity, or quality of life. The review excluded rcts that compared il-2 with surgery or radiotherapy. Six rcts were included in the review. Across these trials, 1098 eligible patients were randomized. None of the trials was placebo-controlled. All of the trials assessed il-2 in combination with other agents, and two of the three-arm trials also included a single-agent il-2 arm. Four trials evaluated subcutaneous il-2, and two trials evaluated intravenous administration at a dose of 18×10^6^ IU/m^2^.

In the five trials that reported objective response rates, the overall weighted objective response rates for il-2–based regimens as compared with regimens that were not based on il-2 were 13.3% (range: 9%–39%) and 5.3% (range: 0%–20%; *p* ≤ 0.001) respectively. Pooled analysis of 1-year mortality data showed no statistically significant difference between il-2–based regimens and non–il-2 controls (risk ratio: 0.94; 95% ci: 0.67 to 1.30; *p* = 0.69).

A Cochrane systematic review 5,6 also reviewed il-2 with a range of other immunotherapies. Results from that meta-analysis also showed no differences between il-2 regimens and non–il-2 regimens in both 1-year mortality and remission rates.

Overall, il-2–containing regimens appeared more toxic than did non–il-2 regimens, but the side effects were described as moderately- to well-tolerated by most patients in most of the trials. The most common grades 3 and 4 toxicities associated with il-2–based treatment were fever, chills, malaise, anorexia, oliguria, nausea or vomiting (or both), diarrhea, skin rash or allergies, hypotension, pulmonary distress, and central nervous system and cardiac toxicity.

### 2.4 High-Dose Interleukin-2

High-dose il-2 has been defined as il-2 administered as an intravenous bolus of at least 600,000IU/kg every 8 hours, or a dose exceeding 65×10^6^ IU/m^2^ daily.

The review by Hotte *et al.* and the Cochrane review 5,6,19 did not identify any randomized phase iii trials comparing high-dose intravenous il-2 with a non–il-2 control or placebo; thus the true clinical effectiveness of the treatment remains unclear. For this reason, it is impossible to recommend use of high-dose intravenous il-2 outside of clinical trials or investigative settings.

A published series by Fisher and colleagues combining data from seven nonrandomized single-arm phase ii trials suggests that approximately 9% of patients can experience complete and long-lasting remissions with high-dose il-2. The lack of control subjects makes interpretation of these data difficult. Proper patient selection is important given the toxicity associated with high-dose il-2 therapy, and some attempts have been made with some success to identify tissue markers that predict for better response. Atkins *et al.* 20 found that expression levels of carbonic anhydrase ix (caix) in tumour correlate with response. Survival was also significantly longer in patients whose tumour specimens stained highly for caix relative to patients whose tumour specimens expressed low levels of caix. Survival for more than 5 years was seen only in patients whose tumour specimens highly expressed caix.

### 2.5 Combination Regimens of Interferon and Interleukin-2

The Cochrane systematic review 5,6 included two trials comparing il-2 plus infα with infα alone. In both of those trials, il-2 combined with infα was associated with a statistically significant improvement in response rates as compared with infα alone, but that response did not translate into an improvement in survival at 1year.

In a trial by Atzpodien and colleagues 21, median survival was longer for patients treated with a combination of il-2 and infα than with either 5-fluorouacil [5-fu (25 months; *p* = 0.04)] or 13*-cis-*retinoic acid (27 months; *p* = 0.02) than for patients treated with a combination of infα2a and vinblastine (16 months). In an earlier trial by the same group 22, a statistically significantly longer median survival was observed with il-2 combined with infα and 5-fu than with tamoxifen (24 months vs. 13 months; *p* = 0.03). Furthermore, median pfs at 1 year was significantly longer for patients treated with il-2 combined with infα2a (20 months) than for patients treated with single-agent il-2 (15 months; *p* = 0.01) or single-agent infα2a (12 months; *p* = 0.01).

### 2.6 Interferon Compared with Interleukin-2

The Cochrane systematic review 5,6 also analyzed trials comparing il-2–based regimens with infα alone. The il-2–based immunotherapies were not observed to be superior to infα, but il-2–containing regimens were associated with greater toxicity.

### 2.7 Interferon Combined with Inhibitors of Angiogenesis

Many of the new anti-angiogenic agents being studied in patients with rcc have been compared with infs, either inf as a single agent or the anti-angiogenic in combination with inf. Given that studies evaluating anti-angiogenic agents alone in comparison with inf alone are being reviewed elsewhere in this issue of *Current Oncology,* we concentrate here on studies combining inf and an anti-angiogenic agent.

#### 2.7.1 Bevacizumab

In two large rcts 23,24, the combination of bevacizumab and inf was associated with significantly longer pfs than was infα alone. Pooling the pfs data from the two trials in a meta-analysis produced a hr of 0.68 (95% ci: 0.60 to 0.76; *p* < 0.00001), which represents a 32% reduction in the risk of progression or death with combination therapy. Combination therapy was associated with more grades 3 and 4 adverse events and treatment discontinuations, but in both treatment arms, the most frequently reported grade 3 or 4 effects were infα-associated toxicity. Deaths resulting from adverse effects were reported with combination (*n* = 8) and with control (*n* = 7) therapy, and three of those deaths were possibly attributable to treatment with bevacizumab. Notably, no rct of bevacizumab as a single agent has yet to be reported, and bevacizumab should therefore be used in combination only with inf at the present time.

#### 2.7.2 Temsirolimus

One large trial 25 of temsirolimus that included only poor-risk rcc patients compared temsirolimus alone with temsirolimus plus infα and with infα alone. The authors reported longer os with single-agent temsirolimus than with single-agent infα (median: 10.9 months vs. 7.3 months; hr: 0.73; 95% ci: 0.58 to 0.92; *p* = 0.008). No survival benefit was observed in patients treated with the combination of temsirolimus and infα, but toxicity was increased. Median pfs was also longer in patients treated with temsirolimus, either alone or in combination with infα. Temsirolimus-based regimens were associated with significantly more grades 3 and 4 anemia, neutropenia, and thrombocytopenia; however, in general, temsirolimus alone was better tolerated than was any treatment arm that contained infα.

#### 2.7.3 Thalidomide

One trial 26 (*n* = 342) compared the combination of thalidomide and infα with infα alone. No difference in os was observed and a 1-month improvement in pfs was seen with combination treatment (3.8 months vs. 2.8 months; *p* = 0.04). Based on these modest improvements and the considerable toxicity of thalidomide, this treatment should not be routinely used in patients with rcc.

## 3. CONCLUSIONS

For patients with inoperable locally advanced or metastatic rcc, results from recent rcts indicate that anti-angiogenic agents are superior to infα alone and are therefore recommended as the preferred treatment option as single agents or in combination with inf. In circumstances in which targeted therapies cannot be used, single-agent infα may still occasionally be used as a treatment option, given that it has been shown to improve survival and disease control. The benefits of combined immunotherapy (with or without chemotherapy) over infα alone are unclear, and such combinations should therefore be used only in the context of clinical trials. Common side effects of infα include anorexia, fatigue, nausea and vomiting, dry mouth, shivering, and mood changes. The data are still insufficient to support the routine use of high-dose intravenous il-2 therapy outside of a clinical trial, and the toxicity of this treatment warrants its administration in specialized centers.

Although cytoreductive therapy has been evaluated in combination with inf therapy and has been associated with improved survival, its role in combination with anti-angiogenic agents remains to be established.
